# Self-reported lifestyle behaviours in families with an increased risk for type 2 diabetes across six European countries: a cross-sectional analysis from the Feel4Diabetes-study

**DOI:** 10.1186/s12902-022-01115-2

**Published:** 2022-08-24

**Authors:** Marieke De Craemer, Vicky Van Stappen, Ruben Brondeel, Violeta Iotova, Nevena Chakarova, Imre Rurik, Jaana Lindström, Jemina Kivelä, Luis Alberto Moreno, Christina Mavrogianni, Yannis Manios, Greet Cardon

**Affiliations:** 1 Department of Rehabilitation Sciences, Ghent University, Corneel Heymanslaan 10, 9000 Ghent, Belgium; 2 Research Foundation Flanders (FWO), Egmontstraat 5, 1000 Brussels, Belgium; 3 Movement and Sports Sciences, Ghent University, 9000 Ghent, Belgium; 4 Sciensano, 1050 Brussels, Belgium; 5 Clinic of Paediatric Endocrinology, Medical University Varna, 9002 Varna, Bulgaria; 6 Clinical Center of Endocrinology, Medical University of Sofia, 1431 Sofia, Bulgaria; 7 Debreceni Egyetem (UoD), University of Debrecen, 4002 Debrecen, Hungary; 8 Finnish Institute for Health and Welfare, 00271 Helsinki, Finland; 9 Growth, Exercise, Nutrition and Development (GENUD), University of Zaragoza, 50009 Saragossa, Spain; 10 Department of Nutrition and Dietetics, School of Health Science & Education, Harokopio University, 176 76 Athens, Greece; 11 Institute of Agri-Food and Life Sciences, Hellenic Mediterranean University Research Centre, Heraklion, Greece

**Keywords:** Children, Parents, Physical activity, Sedentary behaviour, Dietary behaviours, Vulnerable

## Abstract

**Background:**

A healthy lifestyle decreases the risk of developing type 2 diabetes mellitus. The current cross-sectional study aimed to describe self-reported lifestyle behaviours and compare them to current health guidelines in European Feel4Diabetes-families at risk for developing type 2 diabetes across six countries (Belgium, Finland, Spain, Greece, Hungary and Bulgaria).

**Methods:**

Parents and their children were recruited through primary schools located in low socio-economic status areas. Parents filled out the FINDRISC-questionnaire (eight items questioning age, Body Mass Index, waist circumference, PA, daily consumption of fruit, berries or vegetables, history of antihypertensive drug treatment, history of high blood glucose and family history of diabetes), which was used for the risk assessment of the family. Sociodemographic factors and several lifestyle behaviours (physical activity, sedentary behaviour, water consumption, fruit and vegetable consumption, soft drink consumption, sweets consumption, snack consumption, breakfast consumption) of both adults and children were assessed by parental questionnaires. Multilevel regression analyses were conducted to investigate families’ lifestyle behaviours, to compare these levels to health guidelines and to assess potential differences between the countries. Analyses were controlled for age, sex and socio-economic status.

**Results:**

Most Feel4Diabetes-families at risk (parents and their children) did not comply with the guidelines regarding healthy behaviours, set by the WHO, European or national authorities. Less than half of parents and children complied with the physical activity guidelines, less than 15% of them complied with the fruit and vegetable guideline, and only 40% of the children met the recommendations of five glasses of water per day. Clear differences in lifestyle behaviours in Feel4Diabetes-families at risk exist between the countries.

**Conclusions:**

Countries are highly recommended to invest in policy initiatives to counter unhealthy lifestyle behaviours in families at risk for type 2 diabetes development, taking into account country-specific needs. For future research it is of great importance to focus on families at risk in order to counter the development of type 2 diabetes and reduce health inequity.

**Supplementary Information:**

The online version contains supplementary material available at 10.1186/s12902-022-01115-2.

## Introduction

Diabetes is a serious public health problem in Europe. The International Diabetes Federation (IDF) stated that diabetes affects 59.3 million European adults (8.9% of the population) aged 20–79 years—of which type 2 diabetes is the most common type—and the prevalence is estimated to rise to 68.1 million by 2045 [1]. Type 2 diabetes mellitus is a chronic disease characterized by hyperglycemia which eventually causes macro- (*e.g.,* ischemia, stroke) and microvascular complications (*i.e.,* neuropathy, retinopathy, nephropathy) (https://www.idf.org/aboutdiabetes/what-is-diabetes/facts-figures.html) [2]. Although only a limited amount of information is available about the prevalence of type 2 diabetes in European children (6–18 years), type 2 diabetes is also increasingly diagnosed among this young population (e.g., rate ratio of 1.35 in the UK) [3–6]. The increased prevalence of type 2 diabetes in both children and adults results in a high cost spent on the treatment and management of diabetes and related complications [1] with a worldwide cost of 966 billion dollar in 2021 (https://www.idf.org/aboutdiabetes/what-is-diabetes/facts-figures.html). Therefore, efforts are needed to tackle this problem in both children and adults.

Type 2 diabetes is highly preventable through adopting a healthy lifestyle. Several lifestyle behaviours contribute to the development of type 2 diabetes, including insufficient levels of physical activity (PA), high levels of sedentary behaviour (SB) and unhealthy dietary behaviours [7]. Guidelines and recommendations for these behaviours have been developed for children and adults. PA guidelines set by the World Health Organisation (WHO) recommend children to engage in at least 60 min of moderate-to-vigorous PA (MVPA) per day [8] and adults to engage in at least 150 min of MVPA per week (corresponding to at least 30 min of MVPA during 5 days) [9]. For SB, studies and national guidelines recommend for both children and adults to minimise the amount of time spent in prolonged sitting and to break up long periods of sitting as often as possible [10]. Further, specifically for children it is recommended not to exceed two hours of recreational screen-time per day [11–13]. Finally, the WHO recommends to consume at least 400 g of fruit and vegetables, corresponding to five portions per day (for both children and adults) [14], and to limit the daily consumption of unhealthy snacks and soft drinks (not exceeding WHO recommendations for free sugar, < 10% of total energy intake) [15]. In addition, recommendations based on previous research, suggest to consume breakfast on a daily basis [16] and the European Food Safety Authority recommends a daily water (fluid) intake of 1.6–2.0 L of water per day for girls and women and boys and men respectively [17].

Several European studies have previously investigated the levels of PA, SB and healthy dietary behaviours in primary schoolchildren and adults in a general population. Results indicated that between 5.0% and 47.0% of the European children [18] and between 7.0% and 96.0% of the European adults [19] met the recommended amount of PA per day, depending on assessment methods (i.e., different types of questionnaires, accelerometers) and country [18, 19]. Furthermore, European adults reported a sitting time of 309 min/day on weekdays [20] and 25% of the girls and 33% of the boys exceeded the recommended daily screen-time limits [21]. Regarding dietary behaviour, results of the European Energy-project showed that between 12.0 and 51.7% of children and between 11.8 and 56.1% of adults, depending on country and sex, skip breakfast [22]. Furthermore, research has indicated that between 76 and 94% of 11-year-old European children [23] and 86% of adults [24] did not reach the recommendations regarding fruit and vegetable intake. Moreover, a study conducted in six European countries showed that young children consumed on average between 0.25 and 0.85 L of water per day (depending on gender and country) [25] and French, Italian and Spanish adults consumed on average between 0.60 and 0.72 L of water per day [26], which is in both children and adults below the recommended amount (1.5 L of water/day in both age groups). Finally, results of the Identification and prevention of Dietary and lifestyle induced health EFfects In Children and infantS (IDEFICS)-study revealed high intakes of total sugars and foods and drinks rich in added sugar in European 2-to 10-year-old children [26] and a high percentage (11 – 49%, depending of country) of European adults was found to exceed the WHO recommendation on free sugars intake, considering solely fluids [27].

Engaging in a healthy lifestyle (i.e., sufficient physical activity, low levels of sedentary behaviour and a healthy diet) decreases the risk of developing type 2 diabetes. For example, a systematic review and meta-analysis from Aune et al. (2015) showed that there is strong evidence for an inverse association between physical activity and the risk of type 2 diabetes [28]. Also higher levels of sitting time have been associated with a higher risk for developing type 2 diabetes [29]. Diet furthermore plays an important role with a diet high in glycemic load and with a high glycemic index being associated with a larger risk to develop type 2 diabetes [30], while eating more fruit and vegetables is associated with a lower risk of developing type 2 diabetes [31].

Even more alarming, in vulnerable subpopulations (e.g. children and parents with a low socio-economic status (SES)) higher levels of physical inactivity, high levels of SB and unhealthy dietary patterns have been reported and consequently they have a higher prevalence of overweight, obesity and type 2 diabetes compared to the general population [32, 33]. People from lower socio-economic backgrounds have a higher chance to suffer from worse health and have fewer means to buffer against the negative effects of poor health [34]. Unforeseen adverse health events can be particularly devastating for low SES households because they can disturb employment, and create new household financial requirements [34]. In 2019, it was therefore recommended in the International Diabetes Federation Atlas [1] to prioritize families with a higher risk for developing type 2 diabetes in lifestyle interventions (i.e., families from lower SES). However, to our knowledge, no research has been conducted that investigated the prevalence of the above mentioned lifestyle behaviours specifically in parents at increased risk for developing type 2 diabetes and their children.

Therefore, the aim of the current study was to assess the levels of several lifestyle behaviours (i.e. PA, SB and dietary behaviours) and compare these levels to health guidelines/recommendations in European families (parents and their primary schoolchildren) at risk for developing type 2 diabetes. As differences in prevalence of type 2 diabetes exist between European countries [1, 35], this study also investigated potential differences in lifestyle behaviours in these high-risk families between European countries.

## Methods

### Study background

Within the Feel4Diabetes-project a multi-level intervention was developed aiming to prevent type 2 diabetes in vulnerable families across six European countries (Belgium, Finland, Bulgaria, Hungary, Greece and Spain). These countries represent three socio-economic levels: High-income countries (Belgium and Finland), high-income countries under austerity measures (Greece and Spain) and low-to-middle-income countries (Bulgaria and Hungary). The Feel4Diabetes-intervention targets three main lifestyle behaviours, namely PA, SB and dietary behaviour. The Feel4Diabetes-study focused on all types of families with children on the one hand and on families with an increased risk for developing type 2 diabetes on the other hand. This study uses the baseline data from the population at risk for developing type 2 diabetes. Baseline measurements took place from April until June 2016. More detailed information can be found elsewhere [36]. The Feel4Diabetes study is registered with the clinical trials registry clinicaltrials.gov, ID: 643,708 (date of registration: 20/03/2015; retrospectively registered) (https://clinicaltrials.gov/ct2/show/NCT02393872?term=643708&draw=2&rank=1).

### Procedure

Primary schoolchildren and their parents were recruited via schools located in vulnerable areas. Within low-to-middle income countries (Bulgaria and Hungary), all areas were considered vulnerable; whereas in Belgium, Finland, Greece and Spain recruitment only took place in low SES areas. To select the low SES areas, all municipalities in the selected provinces were divided into tertiles based on socioeconomic indices retrieved from official sources and authorities (i.e. literacy or unemployment rates) [37–, 38, 39, 40]. Municipalities within the highest tertile (i.e. municipalities with the lowest SES indices) were included in the study. Within the selected areas, schools were randomly contacted and in total 236 primary schools (response rate = 40.2%) confirmed their participation in the Feel4Diabetes-study. In January 2016, children from the first three grades of primary school and their parents were invited to participate in the study. By signing the informed consent form and filling out two questionnaires (FINnish Diabetes RIsk Score (FINDRISC)-questionnaire and Energy Balance Related Behaviour (EBRB)-questionnaire), families confirmed their participation. The FINDRISC-questionnaire was used to identify families with an increased risk for developing type 2 diabetes (based on the diabetes risk score – see ‘Measurements’ section). In addition, the high-risk families received two additional questionnaires (i.e. the high-risk questionnaires—one for the child and one for the parent) for a more in-depth evaluation of the behaviours. The parents filled out the demographic and lifestyle-related questions for both their children and themselves. In total, 11,396 families confirmed their participation in the project, of which 4,484 families (39.3%) were identified as high-risk families for developing type 2 diabetes (defined as one or both parents having FINDRISC value denoting moderate or high type 2 diabetes risk)[41]. Finally, height and weight were measured by researchers in the school (children) and local municipality centres or home setting (parents). Measurements were conducted by trained researchers, using standardized protocols and calibrated equipment [42].

### Measurements

Before applying the questionnaire to the main study, its reliability was assessed in a pilot study using a two-way random effect single measure intra-class correlation coefficient (ICC). Reliability addressed the question of how consistent the answers were from one occasion to the next in the same subject. Parents were asked to complete the questionnaire twice, within a 1–2 week interval. The ICCs were classified as excellent (> 0.81), good (0.61–0.50), moderate (0.41–0.60) and poor (< 0.40) [43].

#### Diabetes risk score

The FINDRISC-questionnaire is a validated tool to assess individuals’ risk for developing type 2 diabetes. It includes eight questions on age (< 45 years: 0 points; 45–54 years: 2 points; 55–64 years: 3 points; > 64 years: 4 points), Body Mass Index (< 25 kg/m^2^: 0 points; 25–30: 1 point; > 30: 3 points), waist circumference (men: < 94 cm: 0 points, 94-102 cm: 3 points, > 102 cm: 4 points; women: < 80 cm: 0 points; 80-88 cm: 3 points; > 88 cm: 4 points), PA (≥ 4 h/week: 0 points; < 4 h/week: 2 points), daily consumption of fruit, berries or vegetables (no: 1 point; yes: 0 points), history of antihypertensive drug treatment (no: 0 points; yes: 2 points), history of high blood glucose (no: 0 points; yes: 5 points) and family history of diabetes (no: 0 points; yes, 2^nd^ degree relative: 3 points; yes, 1^st^ degree relative: 5 points) [44]. The total risk score is a sum of the individual questions, and values range from 0 to 26, with higher scores indicating a higher risk for developing type 2 diabetes [45]. A family was selected as a high-risk family if at least one of the parents met the cut-off score of 9 points. Further, the highest FINDRISC-score within the family was assessed based on the highest FINDRISC-score of mother or father, or of both parents.

#### Physical activity

MVPA was measured by the following questions in the EBRB-questionnaire: “On how many days during the last week did you (parent) spend MVPA for a total of at least 30 min per day?” and “On how many days during the last week did your child spend in MVPA for a total of at least 1 h per day?”. This question was asked separately for weekdays and weekend days (ICC range 0.37–0.70). To assess the percentage of children and parents meeting the WHO Europe PA guidelines, the number of days meeting the PA guidelines on weekdays and weekend days were summed. The levels of PA in a whole week were dichotomized into not meeting the WHO guideline [46] (< 7 days a week for children and < 5 days a week for parents) and meeting the guideline (7 days a week for children and ≥ 5 days a week for parents).

#### Sedentary behaviour

Parents’ total sitting time on weekdays in parents and screen-time behaviour during the week in children were assessed in respectively the high-risk questionnaire and the EBRB-questionnaire. Total sitting time in parents was assessed using the question: “During the last 7 days, how many hours did you (parent) spend sitting on a weekday?” (ICC = 0.58). Furthermore, children’s screen-time behaviour was assessed by the following question: “About how many hours per day does your child usually devote to screen-activities (excluding school)?”. This question was asked separately for weekdays and weekend days (ICC = 0.65 and 0.69). Answer options were 0 h/d, < 0.5 h/d, 0.5 h/d to < 1 h/d, 1 h/d to < 2 h/d, 2 h/d to < 3 h/d, 3 h/d to < 4 h/d, 4 h/d to < 5 h/d, 5 h/d to < 6 h/d, 6 h/d to < 7 h/d, 7 h/d to > 7 h/d. Afterwards, these categorical values were recoded into numerical values according to the midpoint method (e.g. 0 h/d was recoded into 0 min/day; < 0.5 h/d was recoded into 15 min per day; 2 h/d to < 3 h/d was recoded into 150 min/day; > 7 h/d was recoded into 450 min/day) and the time spend on screen-time activities on weekdays and weekend days were calculated into a mean score using the following formula: (weekdays*5) + (weekend days*2)/7. To determine the percentage of children meeting the recommendation of less than two hours of recreational screen-time per day (based on current WHO guidelines [46]), the total time spend on screen-time activities were dichotomized into not meeting the guideline (≥ 120 min of screen-time activities per day) and meeting the guideline (< 120 min of screen-time activities per day).

#### Dietary behaviour

Consumption of water, soft drinks and juices containing sugar, fruit and vegetables, unhealthy snacks (sweets and salty snacks/fast-food) and breakfast were assessed in the EBRB-questionnaire. The general question asked in the questionnaire was: “Please indicate how often you (parent) and your child consume: Water, soft drinks and juices containing sugar, fruit/berries (fresh or frozen), fruit and berries (canned or dried), vegetables, sweets, salty snacks/fast-food” (ICC range: 0.37–0.83). Answer options were: Less than 1 per week, 1–2 per week, 3–4 per week, 5–6 per week, 1 or 2 per day, 3 or 4 per day, 5 or 6 per day, more than 6 per day. Afterwards, these categorical values were recoded into numerical values according to the midpoint method (e.g. less than 1 portion per week was recoded into 0.07 portion per day, 5–6 portions per week was recoded into 0.8 portion per day, 3 or 4 portions per day was recoded into 3.5 portions per day). The portion size, defined with a household unit, was provided under the question. The consumption of water and soft drinks/juices containing sugar was expressed in glasses, one glass contains a content of 2.5 dl. One portion of fruit and vegetables equals the content of about 1/2 cup (2.5 dl) or the size of a tennis ball. One portion of sweets equals a chocolate bar, half a cup of sweets, cookies or ice-cream and one portion of salty/snacks fast food equals a small hamburger, a small bag of chips or a slice of pizza. To assess the total consumption of fruit and vegetable per day, the daily consumption of fruit/berries (fresh or frozen), fruit and berries (canned or dried) and vegetables were summed. Further, outliers (defined as values above three standard deviations from the mean) were capped, and reassigned the value of the mean plus three standard deviations, a method conducted within previous research [47]. The daily breakfast consumption was measured by the following questions. “On how many days do you/does your child usually eat breakfast?”. This question was asked separately for weekdays and weekend days (ICC range 0.09–0.28). The amount of days consuming breakfast on weekdays and weekend days were summed. Within the Feel4Diabetes-study, families were recommended to drink at least 5 glasses of water per day. To assess the percentage of parents and children meeting these recommendations, the amount of water consumption was dichotomized into not meeting the recommendation (< 5 glasses of water per day) and meeting the recommendation (≥ 5 glasses of water per day). Further, to assess the percentage of parents and children meeting the WHO guidelines regarding fruit and vegetable consumption (at least 5 portions per day), the amount of fruit and vegetable intake was dichotomized into not meeting the guideline (< 5 portions of fruit and vegetables per day) and meeting the guideline (≥ 5 portions of fruit and vegetables per day). Finally, to assess the percentage of parents and children consuming breakfast on a daily basis (recommendation based on previous research), the breakfast consumption in a whole week was dichotomized into not meeting the guideline (< 7 days a week) and meeting the guideline (7 days a week).

#### Sociodemographic variables

Parents reported their birthdate, sex and educational level (years of education), as well as their child’s birthdate and sex. Age was calculated based on parents’ and children’s birthdate and measurement dates. Family SES was categorised as follows: low (both parents having no higher education i.e. ≤ 14 years of education), medium (at least one of the parents having no higher education),high (both parents having a higher education i.e. > 14 years of education) [48]. In European education systems more than 14 years of education implies attendance of higher education (e.g., bachelor program).

### Statistical analyses

Descriptive statistics for the sample demographics were computed using SPSS statistics 24.0 for Windows (SPSS Inc., Chicago, IL) by mean of one-way ANOVA and crosstabs. Besides, in order to have more insight into the strength of the relationship between parents’ lifestyle behaviours and their FINDRISC-score and between children’s lifestyle behaviours and highest FINDRISC-score within the family a correlation analysis was conducted in SPSS statistics 24.0 for Windows (SPSS Inc., Chicago, IL).

Parents’ and children’s lifestyle behaviours and the percentages of parents and children meeting the recommendations/guidelines across all countries were investigated as dependent variables. In addition, potential differences between countries were examined with the use of multilevel regression analyses using MlwiN 2.28 (Centre for Multilevel Modelling, University of Bristol, UK) since data were clustered. Multilevel modelling (three-level: child; class; school) was used to take clustering of children in classes, in schools into account. All analyses were adjusted for age, sex and SES. For all analyses, statistical significance level was set al *p* < 0.01 to take multiple testing into account. Values are reported as means and standard deviations, or percentages.

## Results

### Descriptive data

In total 2,499 high-risk parents (88.8% mothers/stepmothers, mean age 40.1 ± 5.47 years) and 2,506 children (51.1% girls, mean age 8.1 ± 1.01 years) provided data on the EBRB-Questionnaire at baseline. Parents’ FINDRISC-score ranged between 0 and 22 points, with an average of 9.6 ± 4.56 points. Furthermore, the highest FINDRISC-score within the family ranged between 9 and 24 points, with an average of 12.4 ± 2.94 points. Families were assigned to the high-risk group based the FINDRISC-score of the mother (41.7%), the father (35.3%) or both (22.8%). Descriptive data across all countries, and separately for the six countries can be found in Table 1.

**Table 1:** Descriptive data of the study sample: Children and parents from high-risk families in the Feel4Diabetes-study

**Children**								
	**Belgium** ***N* = 420**	**Finland** ***N* = 354**	**Spain** ***N* = 569**	**Greece** ***N* = 494**	**Bulgaria** ***N* = 408**	**Hungary** ***N* = 261**	**All countries** ***N* = 2,506**	***P*-value**
Mean age (SD) in years	8.0 (0.93)	8.7 (0.95)	7.8 (0.96)	7.8 (0.89)	8.3 (0.93)	8.8 (1.03)	8.1 (1.01)	< 0.001 Gr, Sp < Be < Bu < Fi, Hu
% girls	48.1	53.4	49.9	51.0	50.4	56.3	51.1	0.53
% Overweight/obese (> 25 kg/m^2^)	21.1	26.6	30.5	38.1	25.1	34.6	29.4	< 0.001 Be < Bu, Fi < Sp < Hu < Gr
% low family SES	35.6	27.1	5.7	55.9	18.6	67.2	32.8	< 0.001
% medium family SES	30.1	31.1	28.4	26.7	33.7	18.9	28.6	
% high family SES	34.2	41.8	65.9	17.4	47.8	13.9	38.6	
**Highest FINDRISC score within the family**								
Mean (SD)	11.9 (2.68)	12.6 (2.85)	12.1 (2.87)	12.8 (3.05)	12.5 (3.13)	13.0 (2.90)	12.4 (2.94)	NA
Range [min–max]	[9–22]	[9–21]	[9–21]	[9–22]	[9–24]	[9–22]	[9–24]	
**Parents**								
	**Belgium** ***N* = 420**	**Finland** ***N* = 354**	**Spain** ***N* = 568**	**Greece** ***N* = 594**	**Bulgaria** ***N* = 404**	**Hungary** ***N* = 259**	**All countries** ***N* = 2,449**	***P*-value**
Mean age (SD) in years	38.7 (5.50)	40.1 (5.19)	41.5 (5.13)	41.2 (5.04)	39.3 (4.50)	37.6 (6.40)	40.1 (5.47)	< 0.001 Hu < Be, Fi, Bu < Gr, Sp
% mother/stepmother	86.1	87.3	86.3	90.0	94.7	89.0	88.8	NA
% overweight/obese (> 25 kg/m^2^)	61.6	84.3	72.4	66.2	51.8	83.9	66.4	< 0.001 Bu < Be < Gr < Sp < Fi, Hu
% low individual SES	43.1	30.9	7.8	64.1	24.1	71.3	38.0	< 0.001 Sp < Bu < Fi < Be < Gr < Hu
**Parents’ FINDRISC-score**								
mean (SD)	9.7 (4.22)	10.7 (4.30)	9.6 (4.40)	10.1 (4.46)	7.6 (4.89)	10.3 (4.51)	9.6 (4.56)	NA
Range [min–max]	[0–21]	[0–21]	[0–22]	[0–22]	[0–21]	[0–20]	[0–22]	
% of parents with FINDRISC-	32.1	22.1	32.1	31.0	51.3	28.0	33.1	
score < 9 points								

*Be* Belgium, *Fi* Finland, *Sp* Spain, *Gr* Greece, *Bu* Bulgaria, *Hu* Hungary

Besides, the correlations were investigated between parents’ lifestyle behaviours and their FINDRISC-score (correlation coefficient ranged between 0.037 and 0.098), and between children’s lifestyle behaviours and the highest FINDRISC-score within the family (correlation coefficients ranged between 0.009 and 0.079]. Overall, low correlations were found, both in parents and in children. The correlation coefficients can be found in Additional File 1.

#### Self-reported lifestyle behaviours across and between the participating countries

Descriptive data (means and standard deviations) on all lifestyle behaviors in parents and children can be found in Table 2. Furthermore, within the table, differences between the countries were indicated (*p*-values).

**Table 2:** Lifestyle behaviours for parents and children from high-risk families across six European countries

	Belgium	Finland	Spain	Greece	Bulgaria	Hungary	All countries	*P*-value
Moderate-to-vigorous physical activity								
CHILDREN								
Days per week ≥ 60 min per day (SD)	5.0 (0.08)	5.8 (0.09)	5.2 (0.08)	4.7 (0.08)	5.2 (0.08)	5.8 (0.11)	5.2 (0.04)	< 0.001 Fi, Hu > Sp, Bu > Be, Gr
Meeting guidelines (%)	27.7	40.3	31.2	17.1	27.6	43.3	29.7	< 0.001 Fi, Hu > Be, Sp, Bu > Gr
PARENTS								
Days per week ≥ 30 min (mean ± SD)	4.1 (0.12)	4.3 (0.12)	3.4 (0.10)	3.4 (0.11)	3.7 (0.12)	5.0 (0.15)	3.9 (0.05)	< 0.001 Hu > Be, Fi > Gr, Sp, Bu
Meeting guidelines (%)	48.0	49.3	37.7	37.0	38.1	65.2	43.7	< 0.001 Hu > Be, Fi > Gr, Sp, Bu
Sedentary time								
Screen-time in CHILDREN								
Minutes per day mean (SD)	108.8 (3.24)	113.6 (3.52)	88.6 (2.91)	106.2 (2.93)	118.5 (3.25)	125.7 (4.11)	108.1 (1.36)	< 0.001 Bu, Hu > Be, Fi, Gr > Sp
Meeting guidelines (%)	61.8	62.6	74.6	67.7	58.5	54.9	64.5	< 0.001 Gr, Sp > Be, Fi, Bu, Hu
Sitting time during weekdays in PARENTS								
Hours per day mean (SD)	5.9 (0.22)	5.4 (0.26)	5.0 (0.19)	4.7 (0.18)	5.0 (0.19)	5.3 (0.29)	5.1 (0.09)	< 0.001 Be > Gr, Sp, Bu
Drinking water								
CHILDREN								
Glasses per day	2.9 (0.09)	2.4 (0.09)	4.7 (0.08)	4.5 (0.08)	4.2 (0.08)	4.3 (0.10)	3.9 (0.04)	< 0.001 Gr, Sp > Be, Bu, Hu > Fi
Meeting guidelines (%)	17.7	12.9	55.1	51.1	44.8	51.7	39.8	< 0.001 Gr, Sp, Hu > Be, Fi
PARENTS								
Glasses per day	3.8 (0.10)	4.3 (0.10)	5.0 (0.09)	4.6 (0.09)	4.7 (0.10)	4.7 (0.13)	4.6 (0.04)	< 0.001 Sp, Bu, Hu > Fi, Gr > Be
Meeting guidelines (%)	41.0	50.4	67.2	58.5	60.0	65.3	57.3	< 0.001 Sp, Bu, Hu > Fi, Gr > Be
Fruit and vegetable consumption								
CHILDREN								
Portions per day	2.6 (0.08)	2.5 (0.09)	2.6 (0.7)	2.0 (0.08)	2.8 (0.08)	2.2 (0.11)	2.4 (0.04)	< 0.001 Be, Fi, Sp, Bu > Gr, Hu
Meeting guidelines (%)	8.0	11.2	10.1	6.9	11.9	8.9	9.4	0.03
PARENTS								
Portions per day	2.8 (0.09)	3.0 (0.10)	2.8 (0.08)	2.0 (0.09)	3.0 (0.09)	2.0 (0.12)	2.6 (0.04)	< 0.001 Be, Fi, Sp, Bu > Gr, Hu
Meeting guidelines (%)	14.9	17.7	15.8	7.7	14.1	10.9	13.5	< 0.001 Be, Fi, Sp, Bu > Gr, Hu
Consumption of soft drinks or juices containing sugar								
CHILDREN								
Glasses per day	0.5 (0.05)	0.3 (0.05)	0.3 (0.04)	0.1 (0.04)	0.3 (0.04)	1.2 (0.06)	0.4 (0.02)	< 0.001 Hu > Be, Fi, Sp, Bu > Gr
PARENTS								
Glasses per day	0.5 (0.05)	0.2 (0.05)	0.3 (0.04)	0.1 (0.04)	0.3 (0.05)	1.1 (0.06)	0.4 (0.02)	< 0.001 Hu > Be, Fi, Gr, Sp, Bu
Consumption of sweets								
CHILDREN								
Portions per day	1.1 (0.04)	0.4 (0.05)	0.7 (0.04)	0.7 (0.04)	0.8 (0.04)	1.4 (0.05)	0.8 (0.02)	< 0.001 Hu > Be, Gr, Sp, Bu > Fi
PARENTS								
Portions per day	1.0 (0.04)	0.5 (0.05)	0.6 (0.04)	0.5 (0.04)	0.7 (0.04)	1.1 (0.06)	0.7 (0.02)	< 0.001 Be, Hu > Sp, Bu > Fi, Gr
Consumption of salty snacks, fast food								
CHILDREN								
Portions per day	0.3 (0.04)	0.1 (0.04)	^INVALID^	0.2 (0.04)	0.5 (0.04)	1.0 (0.05)	0.4 (0.02)	< 0.001 Hu > Be, Bu > Fi, Gr
PARENTS								
Portions per day	0.2 (0.03)	0.1 (0.03)	0.2 (0.03)	0.1 (0.03)	0.3 (0.03)	0.6 (0.04)	0.2 (0.01)	< 0.001 Sp > Be, Sp, Bu > Fi, Gr
Breakfast consumption								
CHILDREN								
Days per week	6.4 (0.07)	6.8 (0.07)	6.9 (0.06)	6.4 (0.06)	6.5 (0.06)	6.4 (0.08)	6.6 (0.03)	< 0.001 Fi, Sp > Be, Gr, Bu, Hu
Meeting guidelines (%)	82.5	92.4	95.9	82.9	83.1	82.3	86.9	< 0.001 Fi, Sp > Be, Gr, Bu, Hu
PARENTS								
Days per week	5.9 (0.11)	6.7 (0.12)	6.5 (0.10)	5.0 (0.10)	4.3 (0.11)	5.10 (0.14)	5.60 (0.05)	< 0.001 Fi, Sp > Be, Gr, Hu > Bu
Meeting guidelines (%)	71.8	89.1	85.3	52.9	40.0	56.7	66.5	< 0.001 Fi, Sp > Be, Gr, Hu > Bu

*INVALID* No valid cases because of missing question, *Be* Belgium, *Fi* Finland, *Sp* Spain, *Gr* Greece, *Bu* Bulgaria, *Hu* Hungary

#### Moderate-to-vigorous physical activity

##### *Children*

Across the participating countries, children spent 5.2 days per week being moderately to vigorously physically active for at least 60 min per day and 29.7% of children complied with the PA guideline (being moderate-to-vigorous physically active for at least 60 min, 7 days per week). Significant differences exist between the countries (*p* < 0.001). Children from Hungary and Finland were significantly more physically active (5.8 days/week) compared to the other countries (*p* < 0.001), while children from Greece and Belgium spent the lowest number of days being at least 60 min physically active (respectively 4.7 and 5.0 days/week) (*p* < 0.001). Further, in Hungary and Finland a significantly higher percentage of children (respectively 43.3% and 40.3%) complied with the PA guidelines compared to the other participating countries (*p* < 0.01). On the other hand, the lowest number of children complied with the PA guidelines was found in Greece (17.1%; *p* < 0.01).

##### *Parents*

Overall, parents spent 3.9 days per week being moderate-to-vigorous physically active for at least 30 min per day and in total 43.7% complied with the PA guideline (being moderately to vigorously physically active for at least 30 min, 5 days per week). Significant differences exist between the participating countries (*p* < 0.001). Hungarian parents had the highest number of days in which they were physically active (5.0 days/week) compared to the other countries (*p* < 0.01). In Greece, Spain and Bulgaria, parents had the lowest number of days in which they were physically active (respectively 3.4, 3.4 and 3.7 days/week). The highest percentage of parents complying with the PA guidelines was found in Hungary (64.2%) (*p* < 0.01), while the lowest percentages of parents complying with the guidelines were found in Spain (37.7%), Greece (37.0%) and Bulgaria (38.1%) (*p* < 0.01).

#### Sedentary behaviour

##### *Children*

Children across the participating countries spent on average 108 min per day in front of screens and 64.5% complied with the screen-time guideline (less than 120 min/day). Significant differences exist between the countries. Spanish children spent 89 min in front of screens, which is the lowest amount of time compared to the other participating countries (*p* < 0.001). The highest amounts were found in Hungarian and Bulgarian children (respectively 126 and 119 min/day) (*p* < 0.01). The percentage of children meeting the screen-time guideline ranged between 74.6% in Spanish children and 54.9% in Hungarian children. A significantly higher number of Spanish and Greek children complied with the screen-time guideline compared to the other countries (*p* < 0.001).

##### *Parents*

Overall, parents spent on average 5.1 h in sitting time during the day. Significant differences exist between the participating countries (*p* < 0.001). More specifically, Belgian parents had a significantly higher amount of sitting time (5.9 h/day) compared to Greek (4.7 h/day), Spanish and Bulgarian (5.0 h/day) parents (*p* < 0.01). Furthermore, no differences exist between the other countries.

### Dietary behaviour

#### Drinking water

##### *Children*

Across the countries, children consumed on average 3.9 glasses of water per day (approximately 975 ml/day) and 39.8% complied with the guideline regarding water consumption (drinking at least 5 glasses per day or 1250 ml/day). Significant differences exist between the participating countries (*p* < 0.001). More specifically, Spanish and Greek children consumed the highest amount of water (respectively 4.7 (1175 ml) and 4.5 glasses (1125 ml)/day) compared to the other countries (*p* < 0.01). The lowest amount of water consumption was found in children from Finland, who consumed 2.4 glasses per day (600 ml) (*p* < 0.001). The highest percentages of children meeting the guidelines regarding water consumption were found in Spain (55.1%), Greece (51.1%) and Hungary (51.7%) (*p* < 0.001), while only 12.9% of Finnish children and 17.7% of Belgian children complied with the guideline (*p* < 0.001).

##### *Parents*

Overall, parents consumed on average 4.6 glasses of water per day (1150 ml/day) and 57.3% complied with the guideline regarding water consumption (drinking at least 5 glasses per day). Significant differences exist between the participating countries (*p* < 0.001). In Spain, Hungary and Bulgaria, parents consumed respectively 5.0 (1250 ml), 4.7 (1175 ml) and 4.7 glasses (1175 ml) per day, which is significantly higher compared to the other countries (*p* < 0.01), while Belgian parents consumed the lowest amount of water (3.8 glasses or 950 ml/day) (*p* < 0.001). In Spain, Bulgaria and Hungary, respectively 67.2%, 60.0% and 65.3% of the parents complied with the guidelines of water consumption, which is significantly higher compared to the other countries (*p* < 0.01). The lowest percentage of parents meeting the guideline was found in Belgium (41.0%) (*p* < 0.01).

#### Fruit and vegetable consumption

##### *Children*

Across the countries, children consumed on average 2.4 portions of fruits and vegetables per day and differences were found between the countries (*p* < 0.001). Children in Bulgaria, Spain, Belgium and Finland consumed more fruit and vegetables compared to the other countries (*p* < 0.01), respectively 2.8, 2.6, 2.6 and 2.5 portions fruit and vegetables per day. Greek and Hungarian children consumed the lowest amount of fruit and vegetables per day (respectively 2.0 and 2.2 portions/day). Across the countries 9.4% of children complied with the guideline of at least 5 portions of fruit and vegetables per day. No significant differences were found between the participating countries.

##### *Parents*

Across the participating countries, parents consumed on average 2.6 portions of fruit and vegetables per day and 13.5% complied with the guideline of at least 5 portions of fruit and vegetables per day. Further, significant differences exist between the participating countries (*p* < 0.001). Parents from Finland, Bulgaria, Spain and Belgium consumed the highest proportions of fruit and vegetables per day (respectively 3.0, 3.0, 2.8 and 2.8 portions/day) compared to Hungarian and Greek parents who consumed 2.0 portions of fruit and vegetables per day (*p* < 0.001). The percentage of parents meeting the fruit and vegetable guidelines ranged between 17.7% in Finish parents and 7.7% in Greek parents. In Greece and Hungary (10.9%), a significantly lower proportion of parents complied with guidelines compared to the other countries (*p* < 0.01).

#### Consumption of soft drinks and juices containing sugar

##### *Children*

Children across the participating countries consumed on average 0.4 glasses of soft drinks or juices (which equals 100 ml/day) containing sugar per day and significant differences exist between the countries. In Greece, children consumed the lowest amount of soft drinks or juices containing sugar per day (i.e. 0.1 glasses or 25 ml/day) (*p* < 0.001), while in Hungary, children consumed the highest amount of soft drinks or juices containing sugar per day (i.e. 1.2 glasses or 300 ml/day) (*p* < 0.001).

##### *Parents*

Parents across the participating countries consumed on average 0.4 glasses (100 ml) of soft drinks or juices containing sugar per day and significant differences exist between the countries. Hungarian parents consumed a significantly higher amount of soft drinks or soft drinks containing sugar (1.1 glasses or 275 ml per day) compared to parents in Greece (0.1 glasses or 25 ml/day), Finland (0.2 glasses or 50 ml/day), Spain (0.3 glasses or 75 ml/day), Bulgaria (0.3 glasses or 75 ml/day) and Belgium (0.5 glasses or 125 ml/day) (*p* < 0.001).

#### Consumption of sweets

##### *Children*

Children across the countries consumed on average 0.8 portions of sweets per day and significant differences could be detected between the countries (*p* < 0.001). In Finland, children consumed a significantly lower number of sweets (0.4 portions/day) compared to the other countries (*p* < 0.001). The highest consumption of sweets was found in Hungary, with an average of 1.4 portions per day (*p* < 0.001).

##### *Parents*

Parents across the countries consumed on average 0.7 portions of sweets per day and significant differences exist between the countries (*p* < 0.001). Parents from Finland and Greece consumed the lowest number of sweets per day (0.5 portions/day), while in Hungary (1.1 portions/day) and Belgium (1.0 portions/day), parents consumed the highest number of sweets per day (*p* < 0.001).

#### Consumption of salty snacks and fast food

##### *Children*

Children across the participating countries consumed on average 0.4 portions of salty snacks/fast food per day (i.e., a small hamburger, a small bag of chips or a slice of pizza) and differences exist between the countries (*p* < 0.001). In Finland and Greece, children consumed the lowest number of salty snacks and fast food (respectively 0.1 and 0.2 portions/day) (*p* < 0.01), while in Hungary, children consumed 1.0 portions of salty snacks and fast food, which is the highest number compared to the other countries (*p* < 0.001). From Spanish children, no data are available, due to a question missing in the local survey.

##### *Parents*

Parents across the countries consumed on average 0.3 portions of salty snacks and fast food per day and significant differences were found between the countries (*p* < 0.001). Finnish and Greek parents consumed the lowest number of salty snacks and fast food (respectively 0.1 and 0.1 portions/day) compared to the other countries (*p* < 0.01), while the highest number, 0.6 portions per day was found in Hungarian parents (*p* < 0.001).

#### Breakfast consumption

##### *Children*

Children across the countries consumed breakfast on 6.6 days per week and in total 86.9% complied with the daily breakfast guideline (consuming breakfast, 7 days/week). Significant differences exist between the countries (*p* < 0.001). The highest number of days in which children consumed breakfast were found in Spain (6.9 days/week) and Finland (6.8 days/week), while the lowest numbers were found in Belgium (6.4 days/week), Greece (6.4 days/week), Hungary (6.4 days/week) and Bulgaria (6.5 days/week) (*p* < 0.01). The highest percentages of children complying with the daily breakfast guideline were found in Spain (95.9%) and Finland (92.4%), while the lowest percentages were found in Hungary (82.3%), Belgium (82.5%), Greece (82.9%) and Bulgaria (83.1%) (*p* < 0.001).

##### *Parents*

Overall, parents consumed breakfast on average 5.6 days per week and in total 66.5% of all parents complied with the guideline of daily breakfast consumption. Significant differences exist between the participating countries (*p* < 0.001). The highest numbers of days in which parents consumed breakfast were found in Finland (6.7 days/week) and Spain (6.5 days/week) compared to the other countries (*p* < 0.001). In contrast, Bulgarian parents consumed breakfast on 4.3 days per week, which was significantly lower compared to the other countries (*p* < 0.001). The percentages of parents meeting the breakfast guidelines were the highest in Finland (89.1%) and Spain (85.3%), while the lowest percentage was found in Bulgaria (40.0%) (*p* < 0.001).

A more detailed description (*p*-values) of lifestyle behaviours between the participating countries can be found in Additional File 2.

## Discussion

The aim of the current study was to assess several lifestyle behaviours (PA, SB and dietary behaviours) in families at risk for developing type 2 diabetes, and to compare the behaviours to health guidelines. Further, potential differences in at risk families’ lifestyle behaviours between six Feel4Diabetes European countries were investigated.

Overall, some alarming results were found for both parents and children: less than 30% of the children met the PA guideline, only 40% of the children met the guideline of at least 5 glasses of water per day and less than 10% of the children met the guideline regarding fruit and vegetable intake. In addition, less than 45% of the parents met the PA guideline and only 14% of the parents met the guidelines regarding fruit and vegetable intake. On the other hand, results also showed some more positive results: almost 87% of the children and 68% of the parents met the recommendations regarding daily breakfast consumption, 64% of the children met the guideline of screen-time, and both parents and children managed to keep the consumption of soft drinks or juices containing sugar, sweets, salty snacks/fast food relatively low. These results point out that behaviour change programmes in the selected Feel4Diabetes families at increased risk for type 2 diabetes primarily need to focus on the promotion of PA, fruit and vegetable intake and on drinking water. To obtain the adherence to health guidelines, additional efforts to promote daily breakfast consumption and to limit the consumption of unhealthy snacks seem less necessary in this target group.

Despite research showing that people with normal blood glucose levels generally have healthier behaviours compared to people with prediabetes [49, 50], results of this European sample of families at increased risk seems to be quite comparable with results of studies conducted in the general population. More specifically, similar figures were found for adults and children in meeting the guidelines regarding PA, SB and several dietary behaviours [20, 23, 24, 51, 52]. Less positive outcomes for health behaviours could be expected in families at increased risk to develop type 2 diabetes, since they have been selected based on the FINDRISC-questionnaire, which includes questions on these behaviours. However, these questions only account for 3 of 26 points (11.5%) of the total FINDRISC-score, so they only minimally influence the total FINDRISC-score. Further, results showed that the correlations between parents’ lifestyle behaviours and their FINDRISC-score, and the correlations between children’s lifestyle behaviours and families’ FINDRISC-score are overall weak. This is actually not surprising since the prediction model to assess the probability to develop type 2 diabetes within 10 year (conducted to develop the FINDRISC-questionnaire) showed that questions on PA and fruit and vegetable consumption did not add much to the predictive power. Even though they had no impact on the score, these two lifestyle behaviours were included in the FINDRISC-score to emphasize the importance of lifestyle behaviours in preventing type 2 diabetes [44]. Despite the comparable lifestyle outcomes between the general population and the at risk population, this study population needs extra attention in future lifestyle interventions in order to prevent the development of type 2 diabetes.

Similar to cross-European health behaviour differences in the general population [48, 53, 54], results of the present study showed clear cross-European differences in several lifestyle behaviours in high risk families. The cross-European differences, found in all included lifestyle behaviours, are possibly due to political, geographical and cultural differences. In our study, Hungarian children and parents and Finnish children at increased risk had significantly higher PA levels compared to similar participants in the other countries (Belgium, Spain, Greece and Bulgaria). This might partially be explained by differences in the total hours of mandatory physical education in primary schools: Hungarian schools provide five hours of physical education per week, whereas schools in the other countries provide 2 to 3 h of physical education per week [53]. Furthermore, countries differ in provided action plans in the health sectors, schools, workplaces to promote PA, and in national awareness campaigns on PA [53]. Regarding SB, parents at risk for developing type 2 diabetes living in Belgium and Finland (Northern and Central European countries) had higher levels of sedentary time compared to Greece, Spain, Bulgaria and Hungary (Southern European countries), which is in line with the study of Bennie et al. (2013), conducted in 32 European countries in a general population [20]. The study of Bennie et al. (2013) reported on the prevalence of self-reported weekday sitting time in almost 28,000 adults (aged 15–98 years) and compared this weekday sitting time between countries [20]. The differences found in our study and in the study of Bennie et al. (2013) might be explained by wealth inequalities, which could influence several domains of SB (i.e. parents’ occupation, transport, leisure-time and household activities), differences in climate, and/or cultural differences; which has also been reported in previous research [20]. Finally, within this study, clear differences were found in water consumption between Northern and Central European countries, compared to Southern European countries, which could also be found in the study of De Craemer et al. (2015), conducted in preschool children in the general population. This might be explained by higher outside temperatures, resulting in a higher thirst to quench [55]. In conclusion, clear cross-European differences exist in health behaviours in families at risk for developing type 2 diabetes, which are similar compared to cross-European differences reported in the general population.

In addition to the provided recommendations for all countries, namely improvement of physical activity, fruit and vegetable intake and water consumption, also country-specific recommendations could be provided for families at risk for developing type 2 diabetes. First, it should be acknowledged that recommendations regarding the increase of water consumption among Finnish children should be put into perspective as these children are recommended to drink low-fat milk during meals and to drink water when thirsty, instead of soft drinks and juices containing sugar [56]. This fact might explain the low water intake among Finnish children. Within the current study, no data is available on the consumption of low-fat milk. In Belgium, in addition to the general recommendations, it is recommended to reduce children’s screen-time and parents’ total sitting time and to decrease the consumption of soft drinks and juices containing sugar, and sweets among both parents and children. In Finland, additional improvements are needed in children’s screen-time activities and in parents’ total sitting time. In Spain, in addition to the general recommendations, improvements are needed in the consumption of sweets in both parents and children. In Greece, it is additionally recommended to reduce children’s and parents’ sweet consumption. In Bulgaria, in addition to the general recommendations, a decrease is needed in screen-time activities and in the consumption of sweets among children. Finally, in Hungary additional promotion is needed to reduce sedentary time among children and the consumption of soft drinks or juices containing sugar, sweets, salty snacks/fast food in both parents and children.

Although following these recommendations seems easy, it is not that straightforward in people at risk or people with a lower SES. Implementing public health policies might be a better way to move forward in people at risk. However, no single policy or even one field of policy can eradicate health inequalities [57]. Education, income and occupation are policies for which the link between SES and health is the strongest [57]. Regarding education, health benefits might be found in encouraging more years of schooling and supporting early childhood education [57]. Regarding income, a higher income provides the resources for health care, but also for better schooling, nutrition, housing, and recreation [57]. Finally, occupation is a more complex variable but it can affect health as well by means of threat of unemployment and job insecurity [57]. Since it is likely that SES disparities will increase, health promotion efforts should be targeted at the poor [57]. Education for example might also help to stress the importance to parents that parents’ behaviours have an influence on their child’s behaviours. A study by Latomme et al. (2019) showed that higher levels of physical activity performed by fathers is positively associated with physical activity of the child. The same was found for screen time of the father and screen time of the child. Higher levels of fathers’ screen time was associated with higher levels of children’s screen time [58]. Similar results were found in the study of Garriguet et al. (2017) also looking at physical activity and sedentary behaviour of parents and how this is associated with physical activity and sedentary behaviour of their children [59]. A study of Scaglioni et al. (2018) showed similar results for nutrition since parental dietary patterns seem to have the largest influence on children [60]. Parents are the persons that are responsible for the home food environment, they influence how a child thinks about food, and how a child starts building his/her own food preferences and behaviours [60]. Therefore, future interventions should focus on the relationship between parents’ and children’s behaviours and take these into account.

For future research, it is recommended to deliver effective universal interventions (targeting the general population), with a special focus on targeted interventions in individuals/families with an increased risk for developing type 2 diabetes – known as proportionate universalism – in order to reduce health inequity [61]. Second, the FINDRISC-questionnaire, used as a tool to select families at risk for developing type 2 diabetes, is a unique and promising approach in health research to identify families’ risk for type 2 diabetes. It is a fast, inexpensive way to raise awareness in parents of their families’ risk [44] and parents have a profound influence on their children’s lifestyle behaviours through their own behaviours [62–64], so lifestyle changes in parents might have a positive effect on children’ lifestyle behaviours. However, it might be interesting to identify other behaviors or factors (e.g. sedentary behaviour) that put families at risk for type 2 diabetes mellitus since the lifestyle behaviors used to identify the at risk families in the current study account for only a small percentage of the variation in the FINDRISC score. Third, within the current study, a low percentage of fathers (11%) engaged in the current study, which is in line with results of the review of Morgan et al. (2017) [65]. Yet it is of great importance to involve them in health research as recent research shows a strong correlation between weight status and lifestyle behaviours of fathers and their children [58, 66–68] and because a growing number of fathers are involved in the health care of their children [69]. Therefore, recruitment strategies in which fathers are explicitly invited to participate, or by communicating the benefits of the research for fathers and their families should be used in further research [70].

A first strength of the study is the large sample across six European countries, representing high income countries, low-to-middle income countries and countries under austerity measures. Another strength is the focus on a population at risk for developing type 2 diabetes. By using the FINDRISC-questionnaire, at risk families could be detected in a fast, inexpensive and non-invasive way but it is important to mention that the questions within the FINDRISC-questionnaire were self-reported. Also families’ lifestyle behaviours were self-reported, potentially causing response bias (e.g. social-desirability bias) [71], which is a limitation of the current study. In addition, the ICC for breakfast consumption was low. Consequently, results related to breakfast consumption should be interpreted with caution. Furthermore, within the Feel4Diabetes study, Bulgaria and Hungary were classified as low-to-middle income countries while the most recent Work Bank classification (2021) classified Bulgaria as upper-middle income country and Hungary as high-income country. Therefore, the results regarding the classification as lower income countries should be interpreted with caution.

## Conclusion

Results revealed that most Feel4Diabetes families at risk (parents and their children) did not comply with the guidelines regarding healthy behaviours set by the WHO, European or national authorities. Especially low compliance was found for fruit and vegetable consumption, drinking water and physical activity, both in children and parents. Despite the fact that no clear differences in lifestyle behaviours exist between the at risk group in the current study and the general population (revealed from previous research), it is still of great importance to focus on this subgroup in future lifestyle interventions in order to counter the development of type 2 diabetes and to reduce health inequity. Countries should investigate in policy initiatives to counter unhealthy lifestyle behaviours in families at risk for developing type 2 diabetes and because of the observed differences between countries, it is recommended to develop interventions taking into account the needs within a specific country.

## Supplementary Information

**Additional file 1.** Correlations between children’s and parents’ lifestyle behaviours and parents’ FINDRISC-score.

**Additional file 2.** Differences in lifestyle behaviours between the participating countries.

## Data Availability

The datasets used and/or analysed during the current study available from the corresponding author on reasonable request.
