# Supplementary material for: Self-reported lifestyle behaviours in families with an increased risk for type 2 diabetes across six European countries: a cross-sectional analysis from the Feel4Diabetes-study
Source: BMC Endocr Disord. 2022 Aug 24;22:213. doi: 10.1186/s12902-022-01115-2 (PMC9404668; doi:10.1186/s12902-022-01115-2)
Supplement: Supplementary file 1 — Additional file 1. Correlations between children’s and parents’ lifestyle behaviours and parents’ FINDRISC-score. [file 12902_2022_1115_MOESM1_ESM.docx]

Additional file 1: Correlations between children’s and parents’ lifestyle behaviours and parents’ FINDRISC-score.

| **Variable** | **Pearson correlation** | **P-value** |
| --- | --- | --- |
| **CHILDREN’S behaviours** | | |
| Waterconsumption – FINDRISC score mother | -0.035 | 0.207 |
| Waterconsumption – FINDRISC score father | 0.079 | <0.001 |
| Fruit and vegetable intake – FINDRISC score mother | -0.057 | 0.004 |
| Fruit and vegetable intake – FINDRISC score father | -0.038 | 0.074 |
| Sweets – FINDRISC score mother | -0.006 | 0.758 |
| Sweets – FINDRISC score father | 0.028 | 0.188 |
| Soft drinks- sugarjuices – FINDRICS score mother | 0.034 | 0.093 |
| Soft drink – sugarjuices – FINDRISC score father | 0.009 | 0.675 |
| Salty snacks/fastfood – FINDRISC score mother | 0.015 | 0.517 |
| Salty snacks/fastfood – FINDRISC score father | 0.063 | 0.009 |
| Breakfast – FINDRISC score mother | -0.031 | 0.120 |
| Breakfast – FINDRISC score father | -0.067 | 0.002 |
| PA – FINDRISC score mother | -0.035 | 0.079 |
| PA – FINDRISC score father | -0.042 | 0.048 |
| Screen-time – FINDRISC score mother | 0.010 | 0.604 |
| Screen-time – FINDRISC score father | 0.022 | 0.304 |
| **PARENTS’ behaviour** | | |
| Waterconsumption – FINDRISC score | -0.048 | 0.021 |
| Fruit and vegetable intake – FINDRISC score | -0.098 | <0.001 |
| Sweets – FINDRISC score | 0.037 | 0.073 |
| Soft drinks- sugarjuices – FINDRIC score | 0.047 | 0.026 |
| Salty snacks/fastfood – FINDRISC score | 0.044 | 0.034 |
| Breakfast – FINDRISC | -0.021 | 0.311 |
| PA – FINDRISC | -0.129 | <0.001 |
| Sitting time- FINDRISC | 0.037 | 0.160 |
