# Supplementary material for: Self-reported lifestyle behaviours in families with an increased risk for type 2 diabetes across six European countries: a cross-sectional analysis from the Feel4Diabetes-study
Source: BMC Endocr Disord. 2022 Aug 24;22:213. doi: 10.1186/s12902-022-01115-2 (PMC9404668; doi:10.1186/s12902-022-01115-2)
Supplement: Supplementary file 2 — Additional file 2. Differences in lifestyle behaviours between the participating countries. [file 12902_2022_1115_MOESM2_ESM.docx]

Additional file 2: Differences in lifestyle behaviours between the participating countries

| Comparison | P-value | |
| --- | --- | --- |
| **SUBJECTIVELY MEASURED MODERATE-TO-VIGOROUS PHYSICAL ACTIVITY** | | |
| Child | | |
|  | Days per week | Meeting the recommendations |
| Belgium-Finland | <0.001 | <0.001 |
| Belgium-Spain | 0.042 | 0.253 |
| Belgium-Greece | 0.036 | 0.001 |
| Belgium-Hungary | <0.001 | <0.001 |
| Belgium-Bulgaria | 0.085 | 0.966 |
| Finland-Spain | <0.001 | 0.005 |
| Finland-Greece | <0.001 | <0.001 |
| Finland-Hungary | 0.955 | 0.434 |
| Finland-Bulgaria | <0.001 | <0.001 |
| Spain-Greece | <0.001 | <0.001 |
| Spain-Hungary | <0.001 | 0.001 |
| Spain-Bulgaria | 0.839 | 0.237 |
| Greece-Hungary | <0.001 | <0.001 |
| Greece-Bulgaria | <0.001 | 0.001 |
| Hungary-Bulgaria | <0.001 | <0.001 |
| Parent | | |
|  | Days per week | Meeting the recommendations |
| Belgium-Finland | 0.250 | 0.736 |
| Belgium-Spain | <0.001 | 0.002 |
| Belgium-Greece | <0.001 | 0.001 |
| Belgium-Hungary | <0.001 | <0.001 |
| Belgium-Bulgaria | 0.013 | 0.006 |
| Finland-Spain | <0.001 | 0.001 |
| Finland-Greece | <0.001 | <0.001 |
| Finland-Hungary | <0.001 | <0.001 |
| Finland-Bulgaria | <0.001 | 0.003 |
| Spain-Greece | 0.904 | 0.889 |
| Spain-Hungary | <0.001 | <0.001 |
| Spain-Bulgaria | 0.045 | 0.803 |
| Greece-Hungary | <0.001 | <0.001 |
| Greece-Bulgaria | 0.036 | 0.706 |
| Hungary-Bulgaria | <0.001 | <0.001 |

| Comparison | P-value | |
| --- | --- | --- |
| **SUBJECTIVELY MEASURED SEDENTARY TIME** | | |
| Child | | |
|  | Screen-activities in hours per day | Meeting the recommendations |
| Belgium-Finland | 0.315 | 0.835 |
| Belgium-Spain | <0.001 | <0.001 |
| Belgium-Greece | 0.553 | 0.073 |
| Belgium-Hungary | 0.001 | 0.075 |
| Belgium-Bulgaria | 0.034 | 0.333 |
| Finland-Spain | <0.001 | <0.001 |
| Finland-Greece | 0.106 | 0.135 |
| Finland-Hungary | 0.025 | 0.057 |
| Finland-Bulgaria | 0.304 | 0.056 |
| Spain-Greece | <0.001 | 0.034 |
| Spain-Hungary | <0.001 | <0.001 |
| Spain-Bulgaria | <0.001 | <0.001 |
| Greece-Hungary | <0.001 | 0.001 |
| Greece-Bulgaria | 0.005 | 0.005 |
| Hungary-Bulgaria | 0.167 | 0.354 |
| Comparison | P-value | |
| Parent | | |
|  | Total sitting time in hours per day |  |
| Belgium-Finland | 0.116 |  |
| Belgium-Spain | <0.001 |  |
| Belgium-Greece | <0.001 |  |
| Belgium-Hungary | 0.058 |  |
| Belgium-Bulgaria | <0.001 |  |
| Finland-Spain | 0.127 |  |
| Finland-Greece | 0.014 |  |
| Finland-Hungary | 0.704 |  |
| Finland-Bulgaria | 0.142 |  |
| Spain-Greece | 0.253 |  |
| Spain-Hungary | 0.317 |  |
| Spain-Bulgaria | 0.967 |  |
| Greece-Hungary | 0.061 |  |
| Greece-Bulgaria | 0.246 |  |
| Hungary-Bulgaria | 0.340 |  |

| Comparison | P-value | |
| --- | --- | --- |
| **WATER CONSUMPTION** | | |
| Child | | |
|  | Cups per day | Meeting recommendations |
| Belgium-Finland | <0.001 | 0.172 |
| Belgium-Spain | <0.001 | <0.001 |
| Belgium-Greece | <0.001 | <0.001 |
| Belgium-Hungary | <0.001 | <0.001 |
| Belgium-Bulgaria | <0.001 | <0.001 |
| Finland-Spain | <0.001 | <0.001 |
| Finland-Greece | <0.001 | <0.001 |
| Finland-Hungary | <0.001 | <0.001 |
| Finland-Bulgaria | <0.001 | <0.001 |
| Spain-Greece | 0.232 | 0.188 |
| Spain-Hungary | 0.003 | 0.347 |
| Spain-Bulgaria | <0.001 | 0.001 |
| Greece-Hungary | 0.046 | 0.886 |
| Greece-Bulgaria | 0.005 | 0.047 |
| Hungary-Bulgaria | 0.671 | 0.070 |
| Parent | | |
|  | Cups per day | Meeting recommendations |
| Belgium-Finland | <0.001 |  |
| Belgium-Spain | <0.001 |  |
| Belgium-Greece | <0.001 |  |
| Belgium-Hungary | <0.001 |  |
| Belgium-Bulgaria | <0.001 |  |
| Finland-Spain | <0.001 |  |
| Finland-Greece | 0.059 |  |
| Finland-Hungary | 0.020 |  |
| Finland-Bulgaria | 0.005 |  |
| Spain-Greece | 0.00 |  |
| Spain-Hungary | 0.054 |  |
| Spain-Bulgaria | 0.037 |  |
| Greece-Hungary | 0.444 |  |
| Greece-Bulgaria | 0.297 |  |
| Hungary-Bulgaria | 0.896 |  |

| Comparison | P-value | |
| --- | --- | --- |
| **FRUIT AND VEGETABLE CONSUMPTION** | | |
| Child | | |
|  | Portions per day | Meeting recommendations |
| Belgium-Finland | 0.327 | 0.136 |
| Belgium-Spain | 0.876 | 0.276 |
| Belgium-Greece | <0.001 | 0.589 |
| Belgium-Hungary | <0.001 | 0.707 |
| Belgium-Bulgaria | 0.094 | 0.057 |
| Finland-Spain | 0.239 | 0.598 |
| Finland-Greece | <0.001 | 0.039 |
| Finland-Hungary | 0.026 | 0.339 |
| Finland-Bulgaria | 0.010 | 0.734 |
| Spain-Greece | <0.001 | 0.087 |
| Spain-Hungary | <0.001 | 0.581 |
| Spain-Bulgaria | 0.107 | 0.355 |
| Greece-Hungary | 0.114 | 0.391 |
| Greece-Bulgaria | <0.001 | 0.012 |
| Hungary-Bulgaria | <0.001 | 0.194 |
| Parent | | |
|  | Portions per day | Meeting recommendations |
| Belgium-Finland | 0.040 | 0.279 |
| 0Belgium-Spain | 0.669 | 0.718 |
| Belgium-Greece | <0.001 | 0.002 |
| Belgium-Hungary | <0.001 | 0.153 |
| Belgium-Bulgaria | 0.123 | 0.745 |
| Finland-Spain | 0.078 | 0.426 |
| Finland-Greece | <0.001 | <0.001 |
| Finland-Hungary | <0.001 | 0.019 |
| Finland-Bulgaria | 0.572 | 0.162 |
| Spain-Greece | <0.001 | <0.001 |
| Spain-Hungary | <0.001 | 0.071 |
| Spain-Bulgaria | 0.225 | 0.479 |
| Greece-Hungary | 0.546 | 0.254 |
| Greece-Bulgaria | <0.001 | 0.007 |
| Hungary-Bulgaria | <0.001 | 0.251 |

| Comparison | P-value | |
| --- | --- | --- |
| **SOFT DRINK CONSUMPTION** | | |
| Child | | |
|  | Cups per day |  |
| Belgium-Finland | 0.006 |  |
| Belgium-Spain | 0.001 |  |
| Belgium-Greece | <0.001 |  |
| Belgium-Hungary | <0.001 |  |
| Belgium-Bulgaria | 0.001 |  |
| Finland-Spain | 0.824 |  |
| Finland-Greece | 0.001 |  |
| Finland-Hungary | <0.001 |  |
| Finland-Bulgaria | 0.683 |  |
| Spain-Greece | <0.001 |  |
| Spain-Hungary | <0.001 |  |
| Spain-Bulgaria | 0.827 |  |
| Greece-Hungary | <0.001 |  |
| Greece-Bulgaria | 0.004 |  |
| Hungary-Bulgaria | <0.001 |  |
| Parent | | |
| Belgium-Finland | <0.001 |  |
| Belgium-Spain | <0.001 |  |
| Belgium-Greece | <0.001 |  |
| Belgium-Hungary | <0.001 |  |
| Belgium-Bulgaria | 0.001 |  |
| Finland-Spain | 0.216 |  |
| Finland-Greece | 0.641 |  |
| Finland-Hungary | <0.001 |  |
| Finland-Bulgaria | 0.073 |  |
| Spain-Greece | 0.067 |  |
| Spain-Hungary | <0.001 |  |
| Spain-Bulgaria | 0.500 |  |
| Greece-Hungary | <0.001 |  |
| Greece-Bulgaria | 0.017 |  |
| Hungary-Bulgaria | <0.001 |  |

| Comparison | P-value | |
| --- | --- | --- |
| **CONSUMPTION OF SWEETS** | | |
| Child | | |
|  | Portions per day |  |
| Belgium-Finland | <0.001 |  |
| Belgium-Spain | <0.001 |  |
| Belgium-Greece | <0.001 |  |
| Belgium-Hungary | <0.001 |  |
| Belgium-Bulgaria | <0.001 |  |
| Finland-Spain | <0.001 |  |
| Finland-Greece | <0.001 |  |
| Finland-Hungary | <0.001 |  |
| Finland-Bulgaria | <0.001 |  |
| Spain-Greece | 0.870 |  |
| Spain-Hungary | <0.001 |  |
| Spain-Bulgaria | 0.057 |  |
| Greece-Hungary | <0.001 |  |
| Greece-Bulgaria | 0.043 |  |
| Hungary-Bulgaria | <0.001 |  |
| Parent | | |
| Belgium-Finland | <0.001 |  |
| Belgium-Spain | <0.001 |  |
| Belgium-Greece | <0.001 |  |
| Belgium-Hungary | 0.118 |  |
| Belgium-Bulgaria | <0.001 |  |
| Finland-Spain | 0.005 |  |
| Finland-Greece | 0.389 |  |
| Finland-Hungary | <0.001 |  |
| Finland-Bulgaria | 0.002 |  |
| Spain-Greece | 0.036 |  |
| Spain-Hungary | <0.001 |  |
| Spain-Bulgaria | 0.653 |  |
| Greece-Hungary | <0.001 |  |
| Greece-Bulgaria | 0.016 |  |
| Hungary-Bulgaria | <0.001 |  |

| Comparison | P-value | |
| --- | --- | --- |
| **CONSUMPTION OF SALTY SNACKS/FASTFOOD** | | |
| Child | | |
|  | Portions per day |  |
| Belgium-Finland | 0.002 |  |
| Belgium-Spain | No valid data available |  |
| Belgium-Greece | 0.051 |  |
| Belgium-Hungary | <0.001 |  |
| Belgium-Bulgaria | <0.001 |  |
| Finland-Spain | No valid data available |  |
| Finland-Greece | 0.191 |  |
| Finland-Hungary | <0.001 |  |
| Finland-Bulgaria | <0.001 |  |
| Spain-Greece | No valid data available |  |
| Spain-Hungary | No valid data available |  |
| Spain-Bulgaria | No valid data available |  |
| Greece-Hungary | <0.001 |  |
| Greece-Bulgaria | <0.001 |  |
| Hungary-Bulgaria | <0.001 |  |
| Parent | | |
| Belgium-Finland | 0.001 |  |
| Belgium-Spain | 0.411 |  |
| Belgium-Greece | 0.006 |  |
| Belgium-Hungary | <0.001 |  |
| Belgium-Bulgaria | 0.318 |  |
| Finland-Spain | 0.010 |  |
| Finland-Greece | 0.538 |  |
| Finland-Hungary | <0.001 |  |
| Finland-Bulgaria | <0.001 |  |
| Spain-Greece | 0.034 |  |
| Spain-Hungary | <0.001 |  |
| Spain-Bulgaria | 0.057 |  |
| Greece-Hungary | <0.001 |  |
| Greece-Bulgaria | <0.001 |  |
| Hungary-Bulgaria | <0.001 |  |

| Comparison | P-value | |
| --- | --- | --- |
| **BREAKFAST CONSUMPTION** | | |
| Child | | |
|  | Days per week | Meeting recommendations |
| Belgium-Finland | <0.001 | <0.001 |
| Belgium-Spain | <0.001 | <0.001 |
| Belgium-Greece | 0.605 | 0.834 |
| Belgium-Hungary | 0.821 | 0.945 |
| Belgium-Bulgaria | 0.127 | 0.785 |
| Finland-Spain | 0.419 | 0.145 |
| Finland-Greece | <0.001 | <0.001 |
| Finland-Hungary | <0.001 | <0.001 |
| Finland-Bulgaria | 0.006 | <0.001 |
| Spain-Greece | <0.001 | <0.001 |
| Spain-Hungary | <0.001 | <0.001 |
| Spain-Bulgaria | <0.001 | <0.001 |
| Greece-Hungary | 0.830 | 0.800 |
| Greece-Bulgaria | 0.277 | 0.939 |
| Hungary-Bulgaria | 0.266 | 0.758 |
| Parent | | |
|  | Days per week | Meeting recommendations |
| Belgium- Finland | <0.001 | <0.001 |
| Belgium-Spain | <0.001 | <0.001 |
| Belgium-Greece | <0.001 | <0.001 |
| Belgium-Hungary | <0.001 | <0.001 |
| Belgium-Bulgaria | <0.001 | <0.001 |
| Finland-Spain | 0.091 | 0.233 |
| Finland-Greece | <0.001 | <0.001 |
| Finland-Hungary | <0.001 | <0.001 |
| Finland-Bulgaria | <0.001 | <0.001 |
| Spain-Greece | <0.001 | <0.001 |
| Spain-Hungary | <0.001 | <0.001 |
| Spain-Bulgaria | <0.001 | <0.001 |
| Greece-Hungary | 0.555 | 0.280 |
| Greece-Bulgaria | <0.001 | <0.001 |
| Hungary-Bulgaria | <0.001 | <0.001 |
